# Prolonged fasting promotes systemic inflammation and platelet activation in humans: A medically supervised, water-only fasting and refeeding study

**DOI:** 10.1016/j.molmet.2025.102152

**Published:** 2025-04-21

**Authors:** Serena Commissati, Maria Lastra Cagigas, Andrius Masedunskas, Giovanna Petrucci, Valeria Tosti, Isabella De Ciutiis, Gayathiri Rajakumar, Kristopher M. Kirmess, Matthew R. Meyer, Alan Goldhamer, Brian K. Kennedy, Duaa Hatem, Bianca Rocca, Giovanni Fiorito, Luigi Fontana

**Affiliations:** 1 Department of Medicine, University of Verona, Italy; 2 Charles Perkins Center, Faculty of Medicine and Health, University of Sydney, Sydney, New South Wales, Australia; 3 Section of Pharmacology, Department of Safety and Bioethics, Catholic University School of Medicine, Rome, Italy; 4 Department of Medicine, Washington University School of Medicine, St. Louis, MO, USA; 5 C_2_N Diagnostics, St Louis, MO, USA; 6 TrueNorth Health Center, Santa Rosa, CA, USA; 7 Healthy Longevity Translational Research Programme, Yong Loo Lin School of Medicine, National University of Singapore, Singapore; 8 Centre for Healthy Longevity, National University Health System, Singapore; 9 Departments of Biochemistry and Physiology, Yong Loo Lin School of Medicine, National University of Singapore, Singapore; 10 NeuroFarBa Department, University of Florence, Florence, Italy; 11 Clinical Bioinformatics unit, IRCCS Istituto Giannina Gaslini, Genoa, Italy; 12 MRC-PHE Centre for Environment and Health, Imperial College London, London, UK; 13 Department of Endocrinology, Royal Prince Alfred Hospital, Sydney, Australia

**Keywords:** Prolonged fasting, Proteomics, Cardiometabolic, Inflammation

## Abstract

**Objective:**

Prolonged fasting (PF), defined as abstaining from energy intake for ≥4 consecutive days, has gained interest as a potential health intervention. However, the biological effects of PF on the plasma proteome are not well understood.

**Methods:**

In this study, we investigated the effects of a medically supervised water-only fast (mean duration: 9.8 ± 3.1 days), followed by 5.3 ± 2.4 days of guided refeeding, in 20 middle-aged volunteers (mean age: 52.2 ± 11.8 years; BMI: 28.8 ± 6.4 kg/m^2^).

**Results:**

Fasting resulted in a 7.7% mean weight loss and significant increases in serum beta-hydroxybutyrate (BHB), confirming adherence. Untargeted high-dimensional plasma proteomics (SOMAScan, 1,317 proteins) revealed multiple adaptations to PF, including preservation of skeletal muscle and bone, enhanced lysosomal biogenesis, increased lipid metabolism via PPARα signaling, and reduced amyloid fiber formation. Notably, PF significantly reduced circulating amyloid beta proteins Aβ40 and Aβ42, key components of brain amyloid plaques. In addition, PF induced an acute inflammatory response, characterized by elevated plasma C-reactive protein (CRP), hepcidin, midkine, and interleukin 8 (IL-8), among others. A retrospective cohort analysis of 1,422 individuals undergoing modified fasting confirmed increased CRP levels (from 2.8 ± 0.1 to 4.3 ± 0.2 mg/L). The acute phase response, associated with transforming growth factor (TGF)-β signaling, was accompanied by increased platelet degranulation and upregulation of the complement and coagulation cascade, validated by ELISAs in blood and urine.

**Conclusions:**

While the acute inflammatory response during PF may serve as a transient adaptive mechanism, it raises concerns regarding potential cardiometabolic effects that could persist after refeeding. Further investigation is warranted to elucidate the long-term molecular and clinical implications of PF across diverse populations.

## Highlights

• Prolonged water-only fasting induces weight loss, metabolic ketosis, and enhances lipid metabolism in volunteers.
• Prolonged fasting reduces circulating amyloid beta proteins, a key component of brain amyloid plaques.
• Prolonged fasting triggers inflammation and platelet activation, potentially impacting cardiometabolic health.

## 1 Introduction

Prolonged fasting (PF), defined as abstaining from energy intake for ≥4 consecutive days [1], has been practiced throughout history for cultural, spiritual, and health-related reasons. Recently, it has gained renewed attention as a potential intervention to promote health and longevity by mitigating cellular aging, reducing inflammation, and lowering the risk of cardiovascular disease and cancer [2,3]. However, the systemic biological adaptations to PF and its effects on inflammation remain unclear. Advances in high-throughput proteomics now enable the simultaneous measurement of thousands of plasma proteins with high specificity, providing a unique opportunity to investigate molecular adaptations to fasting and refeeding. Such an approach addresses the limitations of earlier studies, which were constrained to examining only a small number of specific preselected proteins.

In this study, we examined 20 volunteers attending a fasting clinic before, during, and after an average 10-day water-only fast, followed by an average of 5 days of supervised refeeding with a plant-based diet. Using untargeted plasma proteomics with SOMAScan, we identified both potential benefits and drawbacks of PF and refeeding at the molecular level. PF triggered a significant shift in 6.6% of the plasma proteome; however, less than 1% of these proteins remained significantly altered after refeeding, supporting a transient effect. Contrary to our hypothesis, the primary outcome was a significant increase in inflammation and cytokine signaling via TGF-β, confirming that PF elevates inflammation. Additionally, we observed alterations in neutrophil and platelet degranulation, along with well-documented changes in IGF and PPARα signaling, key regulators of growth and lipid metabolism, respectively. Limited nutrient availability activated the PTEN, STAT3, and MAPK pathways, critical signal transducers that regulate cell proliferation in response to external stimuli. Notably, this study is the first to report that PF significantly reduced amyloid fiber formation and lowered circulating amyloid beta proteins Aβ40 and Aβ42, even though it did not affect their ratio. The findings were validated through targeted mass spectrometry and ELISAs in blood and urine samples, as well as in two external cohorts undergoing similar PF regimens. While PF is commonly associated with health benefits such as weight loss, our findings suggest its effects are more complex and multifaceted, with potential physiological benefits and drawbacks that require an individualized approach to fasting interventions.

## 2 Results

We recruited 20 middle-aged volunteers (mean age: 52.2 ± 11.8 years; mean BMI: 28.8 ± 6.4 kg/m^2^), including 11 women and 9 men. Participants were approached by the study team at TrueNorth Health Center, a facility offering medically supervised fasting. The study team operated independently from the Center. Volunteers followed a fasting and refeeding regimen, consisting of an average 9.8 ± 3.1-day water-only fast, followed by an average 5.3 ± 2.4 days of guided refeeding (Methods). Both fasting and refeeding procedures were based on previously established protocols [4,5]. Blood and urine samples were collected between 6 and 8 am to minimize diurnal variability, processed immediately, and stored at −80 °C for analysis. We conducted an untargeted high-dimensional proteomic analysis using SOMAScan, measuring plasma levels of 1,317 proteins [6]. In addition, targeted mass spectrometry and ELISAs were employed to quantify specific biomarkers in blood and urine. The results were compared with two independent datasets of PF and modified fasting in humans, from Pietzner et al. (2024) and Wilhelmi de Toledo et al. (2019).

At baseline, the average body weight of the volunteers was 85.6 ± 25.6 kg in women and 87.9 ± 15.4 kg in men. By the end of the fasting period, participants experienced significant weight loss, with women losing 6.3 ± 1.7 kg and men losing 6.9 ± 2.2 kg (p < 0.0001), corresponding to reductions of 7.6% and 7.8% of baseline body weight, respectively. BMI decreased by an average of 2.2 ± 0.5 kg/m^2^ (p < 0.0001), a fractional decrease of 7.6%, while waist circumference was reduced by 6% (p < 0.0001). These reductions in body weight, BMI, and waist circumference persisted through the refeeding period (Table 1). Mild adverse events were common, including headaches, weakness, fatigue, insomnia, dry mouth, and orthostatic hypotension (Figure S1), prompting the transition to a broth and/or juice fast in six participants. Other adverse events included severe abdominal pain and diarrhea (n = 1), hypokalemia (n = 1), arrhythmias (n = 1), and dizziness and palpitations (n = 1). When feasible, blood and urine samples collected before this transition were used for analysis (Figure S2). Adherence was high, with all participants exhibiting a physiological fasting response, as evidenced by significantly elevated serum beta-hydroxybutyrate (BHB) concentrations (p < 0.0001), which normalized during refeeding (Table 1). The fasting-induced increase in BHB and its normalization with refeeding were significantly correlated with changes in inflammatory markers (midkine and IL-8), metabolic regulators (FGF19, leptin receptor, chemerin, growth hormone receptor), and MAPK signaling, a crucial mediator of cell proliferation (Figure S3).

**Table 1:** Anthropometric measures and cardiometabolic effects of prolonged fasting in humans.

	Baseline Mean	SD	Fasting Mean	SD	P-value (Fasting vs Baseline)	Refeeding Mean	SD	P-value (Refeeding vs Baseline)
Sex, Female (%)	11 (55%)	–	–	–	–	–	–	–
Age (years)	52.2	11.8	–	–	–	–	–	–
Height (cm)	173.4	10.8	–	–	–	–	–	–
Weight (kg)	86.6	20.6	80.0	19.5	<0.0001	80.5	18.9	<0.0001
BMI (kg/m^2^)	28.8	6.4	26.6	6.2	<0.0001	26.7	6.0	<0.0001
Waist (cm)	96.4	12.7	90.6	12.9	<0.0001	90.7	11.7	<0.0001
DPB (mmHg)	72.1	8.7	64.5	6.7	0.0030	67.7	7.3	0.1857
SBP (mmHg)	123.9	10.2	118.1	12.4	0.0301	116.8	12.1	0.0296
β-hydroxybutyrate (mmol/L)	0.6	0.9	5.0	1.0	<0.0001	0.4	0.3	0.6215
Glucose (mg/dL)	85.7	10.4	70.3	10.5	0.0002	92.8	9.7	0.0155
HOMA-IR	1.7	1.5	0.8	1.1	0.0006	2.2	1.4	0.0266
C-reactive protein (mg/dL)	1.7	1.5	3.9	3.8	0.0004	3.4	9.9	0.6542
Total cholesterol (mg/dL)	192.0	33.5	216.6	47.8	0.0327	168.2	34.5	0.0006
HDL cholesterol (mg/dL)	56.8	19.2	47.8	12.3	0.0015	44.6	10.9	0.0007
Triglycerides (mg/dL)	102.8	46.2	125.4	37.4	0.0745	135.2	47.2	0.0008
LDL cholesterol (mg/dL)	108.4	37.4	131.6	55.5	0.0898	96.7	30.4	0.041
Non-HDL cholesterol (mg/dL)	135.3	34.0	168.8	47.9	0.0061	123.6	33.5	0.0523
Total cholesterol:HDL ratio	3.8	1.5	4.8	1.5	0.0024	4.0	1.1	0.6152
ALT (IU/L)	21.9	11.4	35.9	20.3	0.0003	34.4	19.4	0.0006
AST (IU/L)	22.8	8.5	37.6	17.2	<0.0001	31.5	17.9	0.0350

Data expressed as mean ± SD. Statistical significance was calculated using paired, 2-tailed Student's t or Wilcoxon signed rank test for non-normally distributed data. N = 20 participants, except for cholesterol measurements and triglycerides (N = 19). Significance levels are indicated as p-values.

### 2.1 Proteomics adaptations to prolonged fasting in humans

**Figure 1:**
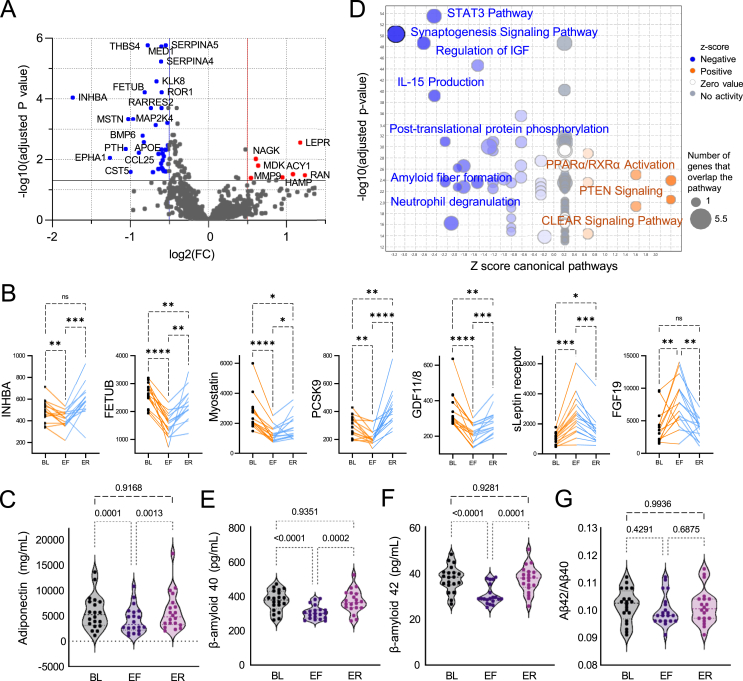
**Proteomics adaptations to prolonged fasting in humans.** (A) Volcano plot of differentially expressed SOMAScan plasma proteins during fasting from N = 15 participants. Significance cut-off adjusted p < 0.05. FC = End of Fasting/Baseline. (B) Individual changes in highlighted proteins from (A) normalized to baseline during fasting and refeeding. Each dot represents protein levels in each participant (N = 15). Adjusted p-value calculated with one-way ANOVA. (C) Absolute levels of plasma adiponectin measured by ELISA across three time points. Each dot represents levels in each participant (N = 20). (D) Volcano plot of differentially enriched canonical pathways in IPA with predicted activation (orange) or inhibition (blue). Input = 1,255 mapped SOMAScan proteins from (A). (E-F-G) Absolute levels of plasma Aβ42, Aβ40, and their ratio measured by IP-LC-MS/MS across three timepoints. N = 20 participants. Statistical analysis is described in the Methods for each analysis. Significance levels are indicated as adjusted p-values, ∗p < 0.05, ∗∗p < 0.01, and ∗∗∗p < 0.001. For all graphs, BL = Baseline, EF = End of Fasting, ER = End of Refeeding.

We found that 6.6% of protein targets (n = 86/1317) exhibited significant changes by the end of fasting (adjusted p < 0.05), with 74 proteins decreasing and 12 increasing (Figure 1A). However, after five days of gradual refeeding, only 12 proteins (<1%) remained significantly altered, indicating that most fasting-induced proteomic changes are transient. The most significantly reduced proteins included key regulators of muscle homeostasis, such as inhibin beta A (INHBA, −3.3 fold, adjusted p = 9.07E-05), myostatin (−2 fold, adjusted p = 0.000466), and GDF11/8 (−1.6 fold, adjusted p = 0.000757), all members of the TGF-β superfamily (Figure 1B). These proteins are vital for muscle regulation, likely reflecting the body's adaptive response to fasting, balancing muscle preservation with tissue repair during nutrient deprivation [7]. Interestingly, inhibiting GDF11 and myostatin has been linked to increased bone density and strength through enhanced osteoblast activity and suppressed osteoclastogenesis [8,9]. Consistent with this, plasma parathyroid hormone (PTH) levels decreased by 2.1-fold (adjusted p = 0.0045), suggesting a compensatory hormonal adjustment during fasting to slow bone loss. Under conditions of energy deprivation, the insulin-sensitizing adipokine adiponectin also decreased from 5643 ± 3282 ng/mL to 4275 ± 2519 ng/mL (p < 0.0001) (Figure 1C).

In contrast, proteins involved in energy, glucose, and bile acid metabolism significantly increased, with PPARα emerging as a key activated pathway (Figures 1D, S4). Regulated by free fatty acids, PPARα drives hepatic lipid metabolism and ketogenesis, a vital fasting adaptation. Another major fasting-activated pathway was the CLEAR (Coordinated Lysosomal Expression and Regulation) network, which governs lysosomal biogenesis and function. This pathway is crucial for autophagy and exocytosis, both essential for cellular maintenance and adaptation to nutrient scarcity. The gut hormone FGF19 also increased 1.8-fold (adjusted p = 0.048) (Figure 1B), playing a crucial role in energy metabolism by regulating bile acid synthesis, enhancing glucose utilization, and promoting hepatocyte proliferation [10]. In addition, the soluble leptin receptor increased significantly (2.2-fold, adjusted p = 0.002), facilitating appetite regulation by regulating leptin bioavailability in the bloodstream [11,12]. The significant increases in FGF19 and the soluble leptin receptor were previously reported in an independent cohort undergoing a 7-day water-only fast by Pietzner et al. (2024) [13]. This comparative cohort of 12 participants experienced an average weight loss of 5.7 ± 0.8 kg, representing a 7.4% reduction in baseline body weight. Comparative proteomics analysis of both cohorts at the 7-day endpoint identified an overlap of 44 significantly decreased and 5 significantly increased proteins, with no discrepancies between studies (Figure S5). Despite methodological differences (Olink vs. SOMAScan), Reactome pathway enrichment analysis revealed that most altered pathways were common to both datasets, particularly those related to neutrophil and platelet degranulation, as well as interleukin, MAPK, and PI3K/AKT signaling (Figure S6). The findings highlight the highly conserved and universal nature of the physiological response to water-only PF.

Additionally, PF was associated with a reduction in synaptogenesis pathways and amyloid fibril formation (Figures 1D, S4). An increasing trend in brain-derived neurotrophic factor (BDNF) levels was observed (1.32-fold, adjusted p = 0.18), which may support previous evidence of fasting's neuroprotective effects [14]. While the predicted decrease in fibrillar formation was not specific to amyloid beta proteins, we hypothesize that it could reflect lower circulating amyloid beta levels. To investigate this, plasma levels of amyloid beta (Aβ) 42, 40, and the Aβ42/Aβ40 ratio - a diagnostic biomarker for brain amyloid plaques [15]- were measured using mass spectrometry [16,17]. Interestingly, PF significantly reduced plasma concentrations of both Aβ42 and Aβ40 (Figure 1E–G), suggesting either a decreased production rate or accelerated degradation of these amyloid peptides during fasting, with levels returning to baseline after refeeding. Even though the reduction in individual Aβ components may have potential beneficial implications for amyloidosis, the Aβ42/Aβ40 ratio, which is the validated biomarker used clinically to identify individuals with brain amyloid plaques [18,19], remained unchanged.

### 2.2 Prolonged fasting increases inflammation

**Figure 2:**
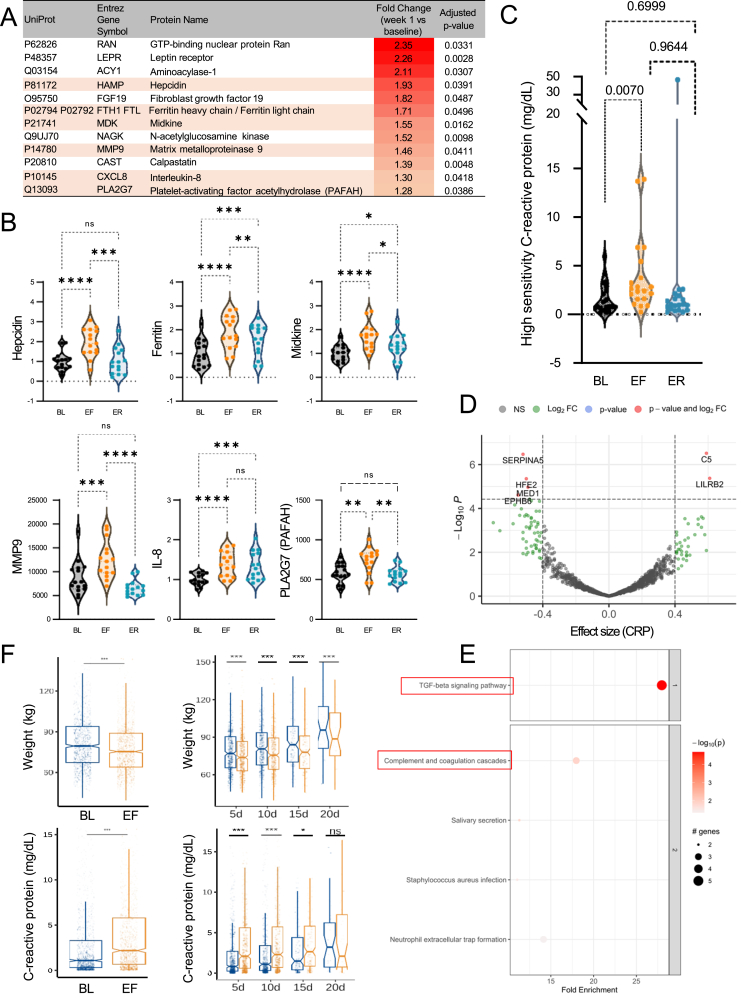
**Prolonged fasting increases inflammation.** (A) All significantly upregulated SOMAScan proteins (n = 12) during fasting normalized to baseline (targets from volcano plot in Figure 2A). Pro-inflammatory proteins are shown with light red background. N = 15 participants. FC = End of Fasting/Baseline (B) Individual changes in 6 inflammatory proteins from panel A during fasting and refeeding. Each dot represents protein levels in each participant. (C) Absolute hsCRP levels in the blood of each participant (N = 20) measured by ELISA across three time points. (D) Significantly correlated proteomic targets to CRP changes during fasting and refeeding (positive effect size = same changes; negative effect size = inverse changes). (E) Significantly enriched KEGG pathways relative to CRP changes. (F) Validation of CRP changes in an independent fasting cohort of 1,422 participants. Measurements of weight and CRP at baseline (BL, blue) and end of fasting (EF, orange) timepoints. The same variables are plotted by fasting length category. 5d = 5 day fast, 10d = 10 day fast, 15d = 15 day fast, 20d = 20 day fast. Median, interquartile range, and outliers are shown, with notches representing the 95% confidence intervals. Statistical analysis is described in the Methods for each analysis. Significance levels are indicated as adjusted p-values, ∗p < 0.05, ∗∗p < 0.01, and ∗∗∗p < 0.001. For all graphs, BL = Baseline, EF = End of Fasting, ER = End of Refeeding.

The primary outcome of our study was inflammation. SOMAScan plasma proteomics analysis revealed significant increases in well-established inflammatory markers, including hepcidin, ferritin, midkine, matrix metalloproteinase 9, IL-8, and platelet-activating factor acetylhydrolase (PAFAH or PLA2G7) (Figure 2A,B). Contrary to our initial hypothesis that fasting would exert an anti-inflammatory effect, PF led to a pronounced 129% increase in circulating high-sensitivity C-reactive protein (hsCRP) levels measured by ELISA (Wilcoxon's p = 0.0004, ANOVA p = 0.0070), with levels returning to baseline after refeeding in all but one participant (Table 1, Figure 2C). The significant rise in hsCRP was positively correlated with C5 (involved in inflammation) and LILRB2 (regulating inflammation and axonal regeneration), and negatively associated with PCI (SERPINA5), hemojuvelin, and MED1 (adipogenesis) (Figure 2D). At the pathway level, CRP was significantly associated with the TGF-β signaling pathway and the complement and coagulation cascade (Figure 2E), suggesting that PF may activate the innate immune response through inflammation. To validate these findings in a broader population, we retrospectively analyzed data from 1,422 individuals who underwent medically supervised modified fasting at the Buchinger-Wilhelmi Clinic in Germany [5]. In this comparative cohort, the average fasting duration was 8.2 ± 0.1 days, with an average weight loss of 4.3 ± 2.0 kg. Notably, 66.6% of participants experienced a significant increase in plasma CRP levels (Figure 2F), confirming the acute inflammatory effect of PF across a larger cohort. Importantly, this increase in CRP was observed regardless of fasting duration (5, 10, 15, or 20 days).

We also observed a significant increase in liver transaminases AST and ALT during fasting, with levels rising by 65% and 64%, respectively, and remaining elevated during refeeding (Table 1). This elevation in liver enzymes, indicative of hepatic stress, was also observed in the Buchinger-Wilhelmi Clinic validation cohort (Fig. S7). The concurrent rise in liver enzymes and inflammatory markers highlights the need for medical monitoring of individuals undergoing PF interventions.

### 2.3 Fasting and refeeding elevate biomarkers of platelet activation and degranulation

**Figure 3:**
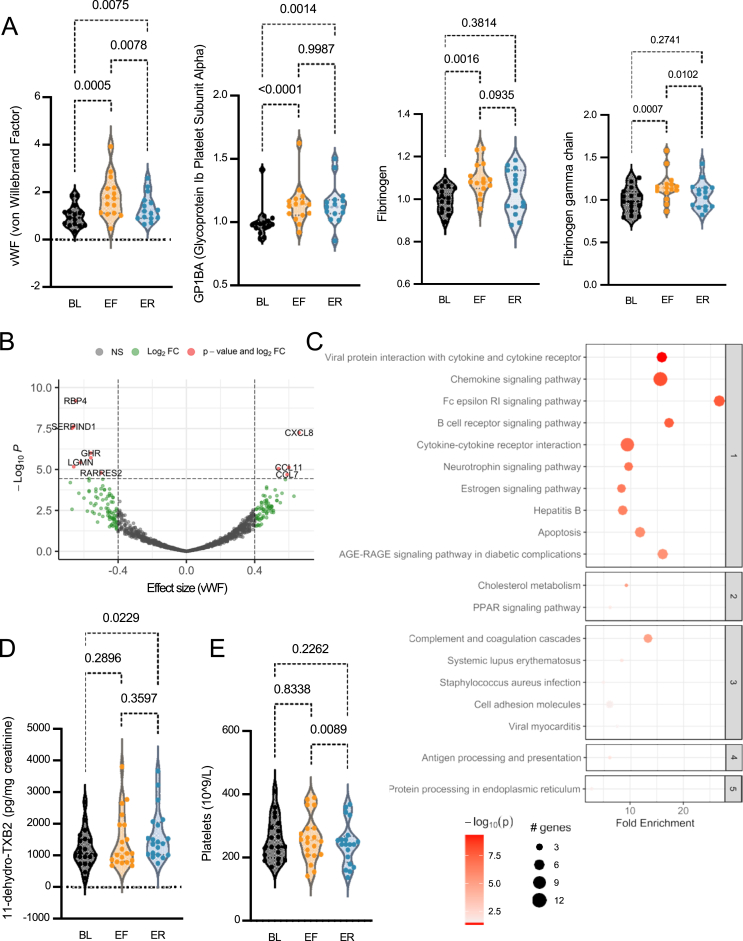
**Fasting and refeeding elevate biomarkers of platelet activation and degranulation.** (A) Individual changes in 4 platelet-associated proteins from SOMAScan normalized to baseline during fasting and refeeding. Each dot represents protein levels in each participant (N = 15). Adjusted p-value calculated with one-way ANOVA. (B) Volcano plot of vWF (effect size) on all 1,317 SOMAScan proteins during combined fasting and refeeding. Significance cut-off adjusted p < 0.01. N = 15 participants. (C) KEGG pathway enrichment analysis for proteins associated with vWF. Fold enrichments in KEGG pathway analysis are shown relative to fold changes for vWF. (D) Absolute TXB2 levels in the urine of each participant (N = 20) across three time points. (E) Absolute platelet counts in the blood of each participant (N = 20) across three time points. Statistical analysis is described in the Methods for each analysis. Significance levels are indicated as adjusted p-values, ∗p < 0.05, ∗∗p < 0.01, and ∗∗∗p < 0.001. For all graphs, BL = Baseline, EF = End of Fasting, ER = End of Refeeding.

An unexpected finding was the observed increase in biomarkers and pathways associated with platelet activity during PF (Figure 3). Reactome pathway enrichment analysis of the SOMAScan data revealed that PF influenced platelet degranulation, a process that facilitates thrombin generation by releasing fibrinogen and von Willebrand factor (vWF) from alpha granules at injury sites [20]. Even though prothrombin levels remained unchanged (1-fold, adjusted p = 0.85), vWF and its receptor, soluble glycoprotein Ib alpha (GP1Bα), were mildly increased (Figure 3A), correlating with elevated chemokines (e.g., IL-8, CCL7, CCL11) (Figure 3B). To confirm platelet degranulation, we measured urinary 11-dehydro-TXB2 levels via ELISA, an enzymatic product in the TXA2/TXB2 pathway primarily derived from activated platelets via cyclooxygenase-1 activity. Surprisingly, 11-dehydro-TXB2 levels rose by 21% during fasting and 36% post-refeeding (Figure 3C), with no change in platelet counts (Figure 3D), indicating that increased degranulation, rather than heightened platelet production, drove the effect [21]. Urinary 11-dehydro-TXB2 is a gold standard biomarker of platelet activation and cardiovascular risk [22]. The Framingham study highlights its predictive value for all-cause mortality, cardiovascular death, and major arterial events [23]. In the ASCEND trial, it was significantly associated with future vascular events in nearly 8,000 diabetic participants [24,25]. Therefore, our findings reveal a PF-induced phenotype characterized by interconnected inflammation and platelet activation, potentially affecting thrombotic risk in individuals with pre-existing conditions.

### 2.4 Prolonged fasting affects cardiometabolic biomarkers

In our study, PF induced significant changes in lipid profiles, including increases in plasma total cholesterol, non-HDL cholesterol, LDL cholesterol, and the total cholesterol/HDL ratio, all of which reversed after refeeding (Table 1). Plasma triglycerides steadily increased, peaking with a 32% rise post-refeeding. Proteomics analysis revealed a significant 1.49-fold reduction in proprotein convertase subtilisin/kexin type 9 (PCSK9) (adjusted p = 0.008) at the end of fasting (Figure 1B). This reduction likely decreased PCSK9 binding to LDL receptors, thereby preventing their degradation in liver cells [26].

In addition to lipid changes, serum glucose levels decreased from 85.7 mg/dL to 70.3 mg/dL (18%) during fasting (Table 1), reflecting the rewiring of whole-body metabolism upon depletion of glucose stores. Similarly, the adipokines chemerin (−1.7-fold, adjusted p = 0.0002) and fetuin B (FETUB) (−1.8-fold, adjusted p = 6.05E-05) were also decreased (Figure 1A, B) [25,26]. During refeeding, glucose levels and HOMA-IR, an indicator of insulin resistance, increased significantly, reflecting enhanced glucose availability with the reintroduction of food.

### 2.5 Fasting and refeeding do not induce systemic changes in oxidation status

While fasting has been suggested to reduce oxidative stress in animal models [27], our proteomic analysis revealed a decrease in superoxide dismutase 3 (SOD3) levels (−1.3-fold, adjusted p = 0.003), an extracellular antioxidant enzyme crucial for redox balance. Additionally, the expected improvements in *in vivo* oxidation status were not observed. Using the validated urinary biomarker of lipid oxidation 8-iso-prostaglandin F2α [25,28], we found a heterogeneous oxidative response to fasting and refeeding. The result suggests that PF does not universally reduce oxidative stress in humans (Fig. S8).

## 3 Discussion

Throughout human evolution, extended periods of food scarcity were common, shaping metabolic flexibility as a survival mechanism. In the context of the global obesity epidemic, fasting has resurged as a popular, sometimes extreme, weight-loss strategy [2,29]. However, the body's adaptations to PF and its potential health effects remain poorly understood. Our study offers a comprehensive proteomic analysis of the responses to PF and refeeding, uncovering both beneficial and potentially detrimental effects. Consistent with previous research [13], we uncovered changes in multiple proteins involved in skeletal muscle and bone homeostasis (INHBA, myostatin, GDF11/8, PTH). Interestingly, exogenous GDF11 has been shown to function as a calorie restriction mimetic in mice, stimulating adiponectin secretion and improving insulin sensitivity [30]. The acute inflammatory response triggered by PF warrants further investigation to clarify its clinical significance, and it is consistent with a clinical study demonstrating that a 10-day water-only fast triggers an inflammatory transcriptional signature in adipose tissue [31]. In our study, inflammation was accompanied by evidence of platelet degranulation, raising concerns as elevated urinary TXB2 has been linked to accelerated atherogenesis and increased cardiovascular risk [24,32]. The inflammatory profile characterized by elevated hsCRP, IL-8, and activation of TGF-β and complement pathways resembles the hallmark features of trained immunity [33], suggesting that PF may serve as an endogenous trigger for this adaptive immune mechanism. However, the immune effects of PF are complex and context-dependent, with evidence supporting fasting-induced modulation of immunosenescence and immune response during immunotherapy [2,[34], [35], [36]].

Prior studies of the adipose tissue transcriptome have linked inflammatory pathways to insulin resistance [[37], [38], [39]]. Consistently, our data show that PF is associated with elevated HOMA-IR. Additionally, increased triglycerides and liver transaminases suggest that prolonged nutrient deprivation, unlike moderate calorie restriction, may disrupt lipoprotein metabolism and liver function [[40], [41], [42], [43]].

Furthermore, this study is the first to demonstrate that PF lowers plasma Aβ42 and Aβ40, key components of amyloid plaques implicated in Alzheimer's disease pathology [44]. These findings suggest that nutrient deprivation alters amyloid precursor protein (APP) expression or processing, influencing either the production or clearance of plasma Aβ42 and Aβ40. Importantly, PF did not affect the Aβ42/40 ratio, a validated biomarker for brain amyloid plaques [18]. This supports the notion that the Aβ42/40 ratio, rather than individual concentrations, is a robust biomarker, as it accounts for inter-individual variability in pre-analytical conditions [45] and presence of comorbidities [46].

Our study has several strengths and limitations. Strengths include the use of two methodologies (mass spectrometry and ELISA) across biological samples (plasma and urine), which yielded consistent findings. Multiple biomarkers were assessed in an untargeted approach, reducing reliance on a single marker, and results were validated in two independent cohorts. Limitations include the single-arm design with a lack of control group, the small sample size, and the variability in fasting and refeeding durations decided by the volunteers.

In summary, our study reveals a multifaceted proteomic response to PF, extending beyond the traditional adipose-centric or energy homeostasis framework. We identified elevated biomarkers related to muscle and bone preservation, reduced amyloid formation, increased inflammation and platelet activity, and lipid metabolism. By conducting a comparative analysis in an independent cohort, we identify a universal signature of the physiological response to water-only PF, observing no differences between studies despite variations in cohort characteristics and methodology. However, we also observed substantial inter-individual variability at the molecular level, emphasizing the need for personalized fasting regimens. In contrast, oxidation status remained unchanged, with no evidence of antioxidant effects. The acute inflammatory response, also observed in an independent cohort, may reflect a positive adaptive mechanism. However, it also raises concerns about a potentially adverse cardiometabolic phenotype, particularly for individuals with thrombotic conditions or unstable atherosclerotic plaques. This mirrors data from exercise interventions, where acute vigorous physical activity can transiently increase cardiovascular risk, particularly in untrained individuals or those with underlying conditions. However, with appropriate progressive training, long-term beneficial adaptations occur, leading to reduced cardiovascular mortality [47]. Unlike exercise, where dose-response relationships and adaptations are well-established, our understanding of how repeated PF bouts impact long-term molecular, metabolic, and clinical outcomes remains limited, highlighting the need for further research.

## CRediT authorship contribution statement

**Serena Commissati:** Methodology, Investigation. **Maria Lastra Cagigas:** Writing – original draft, Visualization, Funding acquisition, Formal analysis, Data curation. **Andrius Masedunskas:** Writing – review & editing, Visualization, Software, Formal analysis. **Giovanna Petrucci:** Methodology, Data curation. **Valeria Tosti:** Methodology, Data curation. **Isabella De Ciutiis:** Writing – review & editing. **Gayathiri Rajakumar:** Writing – review & editing. **Kristopher M. Kirmess:** Writing – review & editing, Methodology. **Matthew R. Meyer:** Writing – review & editing, Methodology. **Alan Goldhamer:** Writing – review & editing, Resources. **Brian K. Kennedy:** Writing – review & editing, Supervision, Resources. **Duaa Hatem:** Methodology. **Bianca Rocca:** Writing – review & editing, Validation, Methodology, Data curation. **Giovanni Fiorito:** Writing – review & editing, Visualization, Validation, Software, Formal analysis. **Luigi Fontana:** Writing – review & editing, Writing – original draft, Supervision, Resources, Project administration, Investigation, Funding acquisition, Conceptualization.

## Declaration of competing interest

A.G. is the founder of TrueNorth Health Center, a private facility that offers medically supervised fasting interventions. K.M.K. and M.R.M. are employed by C_2_N Diagnostics. The other authors report no conflicts of interest. The funding sources were not involved in any form with the findings presented in the study. The article was not commissioned. No author was precluded access to data.

## Data Availability

Data will be made available on request.

## Appendix A Supplementary data

Multimedia component 1 Supplementary Table 1. Inclusion and exclusion criteria. Supplementary Table 2. Medications. Supplementary Figure 1. **Adverse Events (AEs) during fasting and refeeding.** AEs were assessed by a qualified medical practitioner. Supplementary Figure 2. **Sample collection time points.** Blood and urinary samples were collected at baseline (BL), End of Fasting (EF), and End of Refeeding (ER) timepoints. Six participants (IDs 1, 6, 7, 12, 13, 21) switched from water-only fasting to juice and/or broth fasting following medical advice. For these participants, except for ID13, samples for End of Fasting were collected prior to the switch. Three participants (IDs 8, 14, 20) consumed 1-2 juice and/or vegetable broth during the fast. One participant (ID5) consumed juice daily during the fast. Supplementary Figure 3. **BHB association with inflammatory markers and cytokine and MAPK signalling.** Volcano plots of BHB (effect size) on all 1,317 SOMAScan proteomics variables during (A) fasting and (B) combined fasting and refeeding. Significance cut-off adjusted p < 0.01. N = 15 participants. (C-D) KEGG pathway enrichment analysis for proteins associated with BHB. Fold enrichments in KEGG pathway analysis are shown relative to fold changes for BHB. Supplementary Figure 4. **Full list of IPA canonical pathways.** Volcano plot of differentially enriched canonical pathways with predicted activation (orange) or inhibition (blue) using proteomics input from Figure 2A. Supplementary Figure 5. **Comparative proteomics between the SOMAScan dataset and the Olink dataset by Pietzner et al. (2024).** (A) Venn diagram of shared proteins present in both datasets. (B) Significantly altered proteins in each dataset, according to each study’s statistical methods. (C) List of significantly altered shared proteins (44 decreased in blue and 5 increased in red). Supplementary Figure 6. **Comparative Reactome pathway analysis between the SOMAScan dataset and the Olink dataset by Pietzner et al. (2024).** Reactome pathway analysis comparison between (A) SOMAScan and (B) Olink proteomics datasets at fasting day 7 using Uniprot as the reference dataset (accessed October 2024). Pathways are colour matched. BH corrected p-values are shown (grey line). Supplementary Figure 7. **Hepatic markers during fasting by Wilhelmi de Toledo et al. (2019). Validation of ALT and AST changes in an independent fasting cohort of 1,422 participants.** Measurements of ALT and AST at baseline (BL, blue) and end of fasting (EF, orange) timepoints. Median, interquartile range, and outliers are shown, with notches representing the 95% confidence intervals. Statistical analysis is described in the Methods for each analysis. Significance levels are indicated as adjusted p-values, ∗p < 0.05, ∗∗p < 0.01, and ∗∗∗p < 0.001. Supplementary Figure 8. **Oxidative stress and lipid peroxidation status upon prolonged fasting.** (A) SOD3 abundance from SOMAScan proteomics dataset across time points from N = 15 participants. (B) Absolute 8-iso-prostaglandin F2α levels in the urine of each participant (N = 20) across three timepoints. Statistical significance was calculated using Wilcoxon signed rank test for non-normally distributed data for all parameters. Dots represent study participants for all graphs. Significance levels are indicated as p-values. Multimedia component 1
