# Supplementary material for: Prolonged fasting promotes systemic inflammation and platelet activation in humans: A medically supervised, water-only fasting and refeeding study
Source: Mol Metab. 2025 Apr 21;96:102152. doi: 10.1016/j.molmet.2025.102152 (PMC12088818; doi:10.1016/j.molmet.2025.102152)
Supplement: Multimedia component 1 — Supplementary Table 1. Inclusion and exclusion criteria. Supplementary Table 2. Medications. Supplementary Figure 1. Adverse Events (AEs) during fasting and refeeding. AEs were assessed by a qualified medical practitioner. Supplementary Figure 2. Sample collection time points. Blood and urinary samples were collected at baseline (BL), End of Fasting (EF), and End of Refeeding (ER) timepoints. Six participants (IDs 1, 6, 7, 12, 13, 21) switched from water-only fasting to juice and/or broth fasting following medical advice. For these participants, except for ID13, samples for End of Fasting were collected prior to the switch. Three participants (IDs 8, 14, 20) consumed 1-2 juice and/or vegetable broth during the fast. One participant (ID5) consumed juice daily during the fast. Supplementary Figure 3. BHB association with inflammatory markers and cytokine and MAPK signalling. Volcano plots of BHB (effect size) on all 1,317 SOMAScan proteomics variables during (A) fasting and (B) combined fasting and refeeding. Significance cut-off adjusted p < 0.01. N = 15 participants. (C-D) KEGG pathway enrichment analysis for proteins associated with BHB. Fold enrichments in KEGG pathway analysis are shown relative to fold changes for BHB. Supplementary Figure 4. Full list of IPA canonical pathways. Volcano plot of differentially enriched canonical pathways with predicted activation (orange) or inhibition (blue) using proteomics input from Figure 2A. Supplementary Figure 5. Comparative proteomics between the SOMAScan dataset and the Olink dataset by Pietzner et al. (2024). (A) Venn diagram of shared proteins present in both datasets. (B) Significantly altered proteins in each dataset, according to each study’s statistical methods. (C) List of significantly altered shared proteins (44 decreased in blue and 5 increased in red). Supplementary Figure 6. Comparative Reactome pathway analysis between the SOMAScan dataset and the Olink dataset by Pietzner et al. (2024). Reactome pathway an [file mmc1.pdf]

Supplementary Material

Inclusion criteria:

1. Men and women 18 years old and older who have a BMI higher than 20 kg/m2.

Exclusion criteria:

1. Any history of chronic disease processes that according to the investigators and local physicians could interfere with interpretation of the results.
2. Alcoholism, psychiatric problems, life situations that would interfere with study participation and compliance.

Supplementary Table 1

Participant ID	Gender	Age	Medications at baseline?	Symptoms at baseline?	Medications during fasting?	Medications during refeeding?
PF1	F	46	No	Stress symptoms and insomnia (started years ago)	No	No
PF4	M	37	No	None	Fexofenadin 120 mg (1 pill/day for two days)	No
PF5	F	58	No	None	No	No
PF6	F	45	No	Stress symptoms and fatigue (started years ago)	No	No
PF7	M	63	No, stopped meds 4 days before fasting: Hydrochlorothiazide/valsartan 12.5 mg/320 mg/day, allopurinol 300 mg/day, vitamin B12 500 mg/day, vitamin D 2000 IU/day.	None	No	No
PF8	F	45	No, stopped norethindrone 0.35 mg/day 3 days before fasting.	Weakness and headache on baseline day	Dichloralphenazone 100 mg + 325 mg acetaminophen + 65 mg isometheptene, 1 capsule taken	No
PF9	M	55	No, stopped lisinipril/hydrochlorothiaziede 10/12.5 mg 1 tablet daily 1 day before fasting.	Left hip pain during movements (started 10 years ago)	No	No
PF10	F	66	No	None	No	No
PF11	M	42	No, stopped 3 days before fasting a dermatological skin cream (clobetasol 0.05% + ofloxacin 0.75% + ornidazole 2%+ terbinafine 1%) 1-2 times daily.	Stress symptoms (started 10 years ago), current jet lag.	No	No
PF12	F	56	No, stopped 4 days before fasting colecalciferol 50.000 IU once a week.	Fatigue (started 2 months ago after a steroid treatment for anaphylactoid reaction (ivy poison))	No	No
PF13	M	72	No, stopped 2 days before fasting tamsulosin 0,4 mg/daily.	Dizziness and orthostatic hypotension	No	Yes, started tamsulosin 0.4 mg/daily on day 13 due to urinary pain and burning. Symptoms resolved in 2 days.
PF14	M	31	No, stopped 1 day before fasting dehydroepiandrosterone 10 mg/daily.	Headache on baseline day	No	No
PF15	M	32	No	None	No	No
PF16	F	57	No	Headache on baseline day	No	No
PF17	F	63	Yes, conjugated estrogen cream 0.625 mg/g once a day.	Lumbar pain (30 years ago)	Conjugated estrogen cream 0.625 mg/g once a day. Stopped on 2nd fasting day.	No
PF18	F	57	No	None	No	No
PF19	F	56	Yes, CPAP at night (started 15 years ago) and ibuprofen 800 mg due to knee pain a day before fasting.	None	No (continuation with CPAP)	No (continuation with CPAP)
PF20	M	68	No, stopped 4 days before fasting finasteride 1 mg/day, vitamin D 10.000 IU 3 times/week, vitamin B (dose not remembered) 3 times/week, dehydroepiandrosterone 10 mg/daily.	None	No	No
PF21	M	37	No	Stress symptoms (started 2 years ago), insomnia (started 17 years ago)	No	No
PF22	F	58	No	None	No	No

Supplementary Table 2

ID	Gender	Age	Symptoms prior to fasting	Fasting length (days)	Fast broken due to AEs?	Refeeding length (days)	AE first week of fasting	AEs second week of fasting	AEs third week of fasting	AEs during refeeding
**PF1**	F	46	Stress symptoms and insomnia (started years ago)	11	Yes, due to abdominal pain and diarrhea	5	Stress symptoms and insomnia (years ago), headache (4° day), back and leg pain (5°day)	Diarrhea, abdominal pain (11° day) and weakness, stress symptoms and insomnia, back and leg pain	Finished	Stress symptoms and insomnia gone better
**PF4**	M	37	None	15	No	7	None	Fatigue (10°day)	Pollen allergy (15° day) and fatigue	None
**PF5**	F	58	None	10	No	6	Stress symptom, fatigue (years ago), head ache (3° day), orthostatic hypotension and lightheadness (4° day), gastric reflux and sleep problems (5° day)	Neck pain and sleep problems (7°day), weakness	Finished	Weakness, stomach ache, dysuria (1° day), abdominal and breast rash (5° day)
**PF6**	F	45	Stress symptoms and fatigue (started years ago)	22	Yes, switched to juice fasting due to hypokalemia	14	NA	Fog mind (11° day), dizziness (4° day), stress and fatigue	Stress and fatigue, stomach ache→insomnia (15°), head ache (16° day), lightheadness (18° day), hypokalemia (3 mEq/L- 20° day)	None. Fatigue (years ago) gone away 12 days after starting refeeding
**PF7**	M	63	None	21	Yes, switched to broth and juice fasting due to premature atrial and ventricular beats	7	Dry mouth (7° day)	Dry mouth (7° day), weakness and premature atrial and ventricular beats (13° day), diarrhea (15° day)	Premature atrial and ventricular beats (13° day) gone away after 5 days on juice fasting	None. Dry mouth (7° day) gone after 4 refeeding days
**PF8**	F	45	Weakness and headache (started at the baseline)	12	No	5	Dizziness (1° day), head ache and vomit 1 time (2° day), orthostatic hypotension and lightheadness, dry mouth (5° day)	Orthostatic hypotension and lightheadness, dry mouth (5° day), weakness (9°day), back pain (12°day)	Finished	Got better lightheadness, dry mouth (5° day). Eeakness (9°day), back pain (12°day) gone away after 3-4 refeeding days
**PF9**	M	55	Left hip pain during movements (started 10 years ago)	12	No	5	Left hip pain got better, nausea and weakness (4° day), dry mouth (6° day), orthostatic hypotension and lightheadness (5° day)	Left hip pain (years ago), dry mouth (6° day), orthostatic hypotension and lightheadness (5° day)	Finished	Left hip pain (years ago)
**PF10**	F	66	None	14	No	8	Lightheadness (7° day), insomnia (1° day)	Dizziness (15° day), dry mouth (10°day), irritability (14°day), sore throat and fever (37.7°C) (12°day)	Finished	Dizziness (15° day) gone better after started refeedind and started again the last refeeding day, headache (22° day). Sore throat (12°day) gone (20° day)
**PF11**	M	42	Stress symptoms (started 10 years ago), sleep disorders (jet lag – 0° day)	16	Unclear	8	Stress symptoms (started 10 years ago), sleep disorders (jet lag – 0° day), headache and nausea (3° day), dry mouth (5°day), rash on nose and eyelid (5° day), vasovagal syncope with myoclonus during venipuncture (8° day)	stress symptoms (started 10 years ago) gone on the 11° WF, dry mouth (5°day), rash on nose and eyelid (5° day)	Rash on nose and eyelid (5° day) got better, fatigue (15° day), depressed mood and food craving (15° day)	rash on nose and eyelid (5° day) got worse. Dry mouth (5° day), fatigue (15° day), depressed mood (15° day) gone away in 3 days after starting refeeding

ry Table 2.

ID	Gender	Age	Symptoms prior to fasting	Fasting length (days)	Fast broken due to AEs?	Refeeding length (days)	AE first week of fasting	AEs second week of fasting	AEs third week of fasting	AEs during refeeding
**PF12**	F	56	Fatigue (started 2 months ago after a steroide treatment for anaphylactoid reaction (ivy poison)	19	Yes, switched to broth and juice fasting on day 14 and symptoms improved	4	Fatigue (started 2 months ago), dry mouth and lumbar pain (4° day), sleep disorders (6° day), lightheadedness and orthostatic hypotension (3° day)	Fatigue (started 2 months ago) gone worse on 14° day, dry mouth and lumbar pain (4° day), sleep disorders (6° day), lightheadness (3° day)	Fatigue (started 2 months ago) got worse on 19° day	Fatigue (started 2 months ago) got a litte bit better. The patient asked for a short refeeding time (personal reasons)
**PF13**	M	72	Dizziness and orthostatic hypotension	12	Yes, switched to broth and juice fasting on day 11 due to "skip beats" feeling	6	Low concentration (5° day), lightheadness (5° day), acid reflux (6° day), slight stiffness in the shoulders (6° day), nausea (7° day)	Dizzines and "skip beats" feeling (11° day)	Finished	Urinary pain and burning (13° day)
**PF14**	M	31	Head ache (started the day before fasting)	7	No	7	Vomit, nausea and lightheadness (2° day), headache and weakness and orthostatic hypotension (6° day)	Finished	Finished	Orthostatic hypotension (6° day) got better. Headache and weakness (6° day) gone away in few days after starting refeeding
**PF15**	M	32	None	7	No	6	Weakness (6° day)	FInished	Finished	None. Weakness (6° day) gone away in 2 days after starting refeeding
**PF16**	F	57	Head ache (started the day before fasting)	12	No	5	Dry mouth (4° day), weakness (5° day), headache (7° day), nausea, acid reflux (6° day)	Dry mouth (4° day) got worse, weakness (5° day), nausea, acid reflux (6° day) gone away on the 10 day of fasting	Finished	None. Dry mouth (4° day) weakness (5° day) gone away after 3-4 refeeding days
**PF17**	F	63	Lumbar pain (30 years ago)	7	Unclear	6	Lumbar pain (30 years ago) gone away on 2° fasting day, head ache and weakness (3° day and got worse on 6° day), dry mouth and insomnia (4° day), dizziness (6° day), nausea (7° day)	FInished	Finished	Insomnia (4° day), diarrhea on 2° refeeding day. Weakness (3° day) gone away after 6 refeeding days, dry mouth (4° day) after 4 refeeding days; dizziness (6° day) and nausea (7° day) after 2-3 refeeding days
**PF18**	F	57	None	14	No	6	Weakness (3° day), dry mouth (4° day), orthostatic hypotension and dizziness (7° day)	Weakness (3° day), dry mouth (4° day), orthostatic hypotension and dizziness (7° day) gone away on 10° water fasting day	Finished	dry mouth (4° day), weakness (3° day) gone away after 5 refeeding days

ID	Gender	Age	Symptoms prior to fasting	Fasting length (days)	Fast broken due to AEs?	Refeeding length (days)	AE first week of fasting	AEs second week of fasting	AEs third week of fasting	AEs during refeeding
**PF19**	F	56	Continuous positive airway pressure (CPAP) therapy (started 15 years ago)	9	No	5	Nausea (4° day)	Nausea (4° day), lightheadness only one day (9° day)	Finished	Nausea (4° day) gone away after 2 refeeding days
**PF20**	M	68	None	7	No	4	Headache (1 day) spontaneous resolution in one day, nausea (2° day) for 2 days, weakness (1° day) and (3° day) for 1 day each time, back pain (3° day) for one day	Finished	Finished	None
**PF21**	M	37	Stress symptoms (started 2 years ago), insomnia (started 17 years ago)	12	Yes, switched to broth and juice fasting on day 11	5	Insomnia (started 17 years ago), acid reflux (6° day) for 1 day, weakness (8° day)	Insomnia (started 17 years ago) and weakness (8° day) got worse, nausea (10° day) for 1 day, dizziness (11° day) for one day, legs pain (10° day) for 2 days	Finished	Insomnia (started 17 years ago) and weakness (8° day) got better after starting refeeding
**PF22**	F	58	None	10	No	4	Dizziness (2° day) for 1 day, insomnia (4° day) for 1 day, weakness (6° day)	None	Finished	None. Weakness (6° day) gone away after 2 refeeding days

Supplementary Figure 1

ID	Gender	Age	Baseline sample	day 1	day 2	day 3	day 4	day 5	day 6	day 7	day 8	day 9	day 10	day 11	day 12	day 13	day 14	day 15	day 16	day 17	day 18	day 19	day 20	day 21	day 22	day 23	day 24	day 25	day 26	day 27	day 28	day 29	day 30	day 31	day 32	day 33	day 34	day 35	day 36	Fasting days to EF	Refeed days to ER
PF1	F	46	BL	fast	fast	fast	fast	fast	fast	EF	fast	juice	fruit	fast	refeed	refeed	refeed	ER																						7	4
PF4	M	37	BL	fast	fast	fast	fast	fast	fast	fast	fast	fast	fast	fast	fast	fast	fast	EF	refeed	refeed	refeed	refeed	refeed	ER																15	6
PF5	F	58	BL	juice	juice	juice	juice	juice	juice	juice	juice	juice	EF	refeed	refeed	refeed	refeed	ER																						10	5
PF6	F	45	BL	fast	fast	fast	fast	fast	fast	EF	broth	broth	broth	broth	broth	fast	fast	fast	fast	fast	fast	fast	juice	juice	juice	refeed	refeed	refeed	refeed	refeed	refeed	refeed	refeed	refeed	refeed	refeed	refeed	refeed	ER	7	14
PF7	M	63	BL	fast	fast	fast	fast	fast	fast	EF	fast	fast	fast	fast	fast	broth	juice	juice	juice	juice	juice	juice	juice	juice	refeed	refeed	refeed	refeed	refeed	ER										7	6
PF8	F	45	BL	fast	juice	juice	fast	fast	fast	fast	fast	fast	fast	fast	EF	refeed	refeed	refeed	ER																					12	4
PF9	M	55	BL	fast	fast	fast	fast	fast	fast	EF	fast	fast	juice	juice	juice	refeed	refeed	refeed	ER																					7	4
PF10	F	66	BL	fast	fast	fast	fast	fast	fast	fast	fast	fast	fast	fast	fast	fast	EF	refeed	refeed	refeed	refeed	refeed	refeed	ER																14	7
PF11	M	42	BL	fast	fast	fast	fast	fast	fast	fast	juice	juice	fast	fast	fast	fast	fast	fast	EF	refeed	refeed	refeed	refeed	refeed	refeed	ER														16	7
PF12	F	56	BL	fast	fast	fast	fast	fast	fast	fast	EF	fast	fast	fast	fast	fast	broth	broth	broth	broth	juice	juice	refeed	ER																8	2
PF13	M	72	BL	fast	fast	fast	fast	fast	fast	fast	fast	fast	fast	juice	EF	refeed	refeed	refeed	refeed	refeed	ER																			12	6
PF14	M	31	BL	fast	fast	fast	fast	broth	broth	EF	refeed	refeed	refeed	refeed	refeed	ER																								7	6
PF15	M	32	BL	fast	fast	fast	fast	fast	fast	EF	refeed	refeed	refeed	refeed	ER																									7	5
PF16	F	57	BL	fast	fast	fast	fast	fast	fast	fast	fast	fast	fast	fast	EF	refeed	refeed	refeed	ER																					12	4
PF17	F	63	BL	fast	fast	fast	fast	fast	fast	EF	fast	refeed	refeed	refeed	refeed	refeed	ER																							7	6
PF18	F	57	BL	fast	fast	fast	fast	fast	fast	fast	fast	fast	fast	fast	fast	fast	EF	refeed	refeed	refeed	refeed	ER																		14	5
PF19	F	56	BL	fast	fast	fast	fast	fast	fast	fast	fast	EF	refeed	refeed	refeed	ER																								9	4
PF20	M	68	BL	juice	fast	fast	fast	fast	fast	EF	refeed	refeed	ER																											7	3
PF21	M	37	BL	fast	fast	fast	fast	fast	fast	EF	fast	fast	fast	juice	fast	refeed	refeed	refeed	ER																					7	4
PF22	F	58	BL	fast	fast	fast	fast	fast	fast	fast	fast	fast	EF	refeed	refeed	ER																								10	3
Mean																																								9.8	5.3
SD																																								3.1	2.4

Supplementary Figure 2

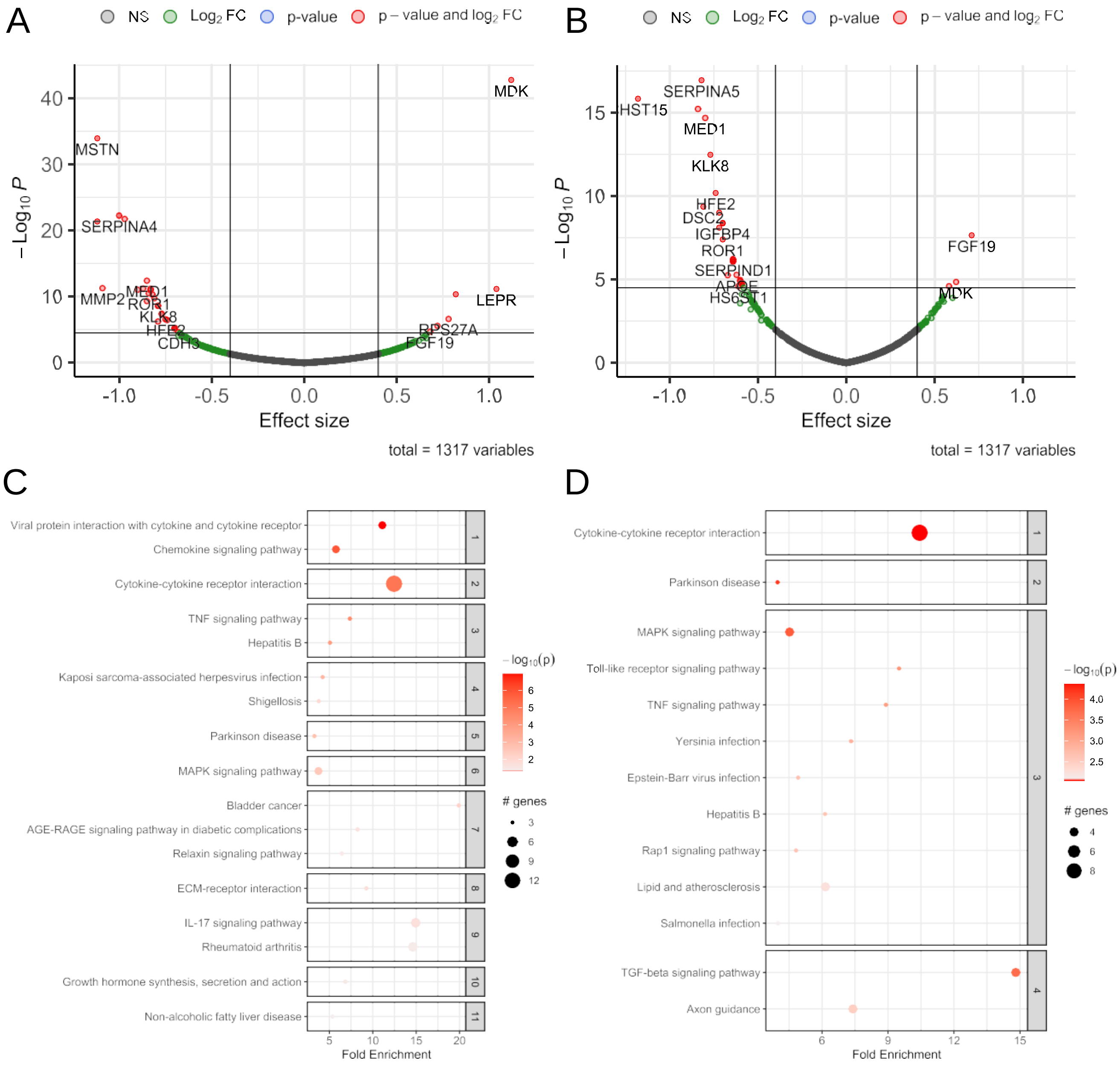

Supplementary Figure 3

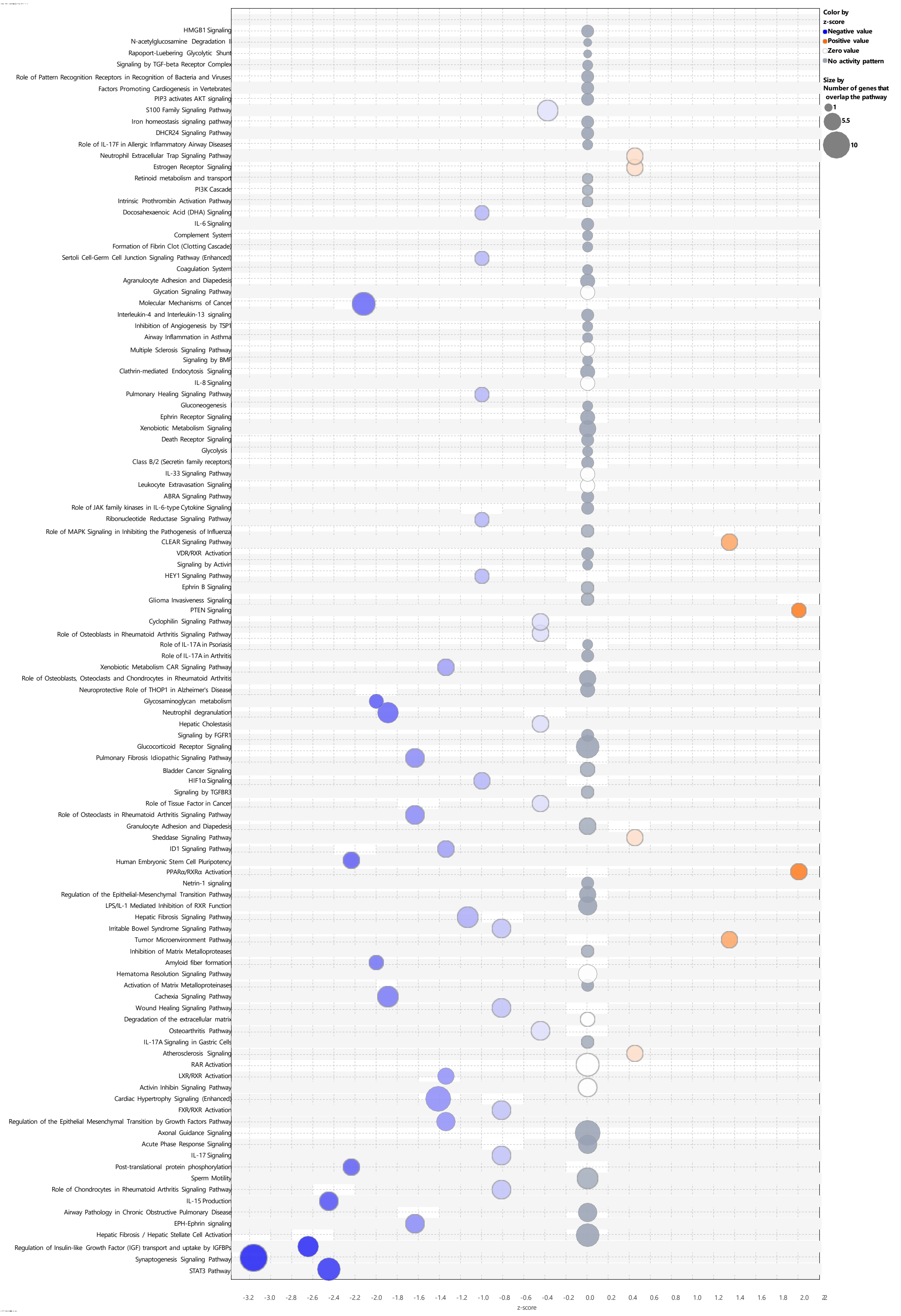

Supplementary Figure 4

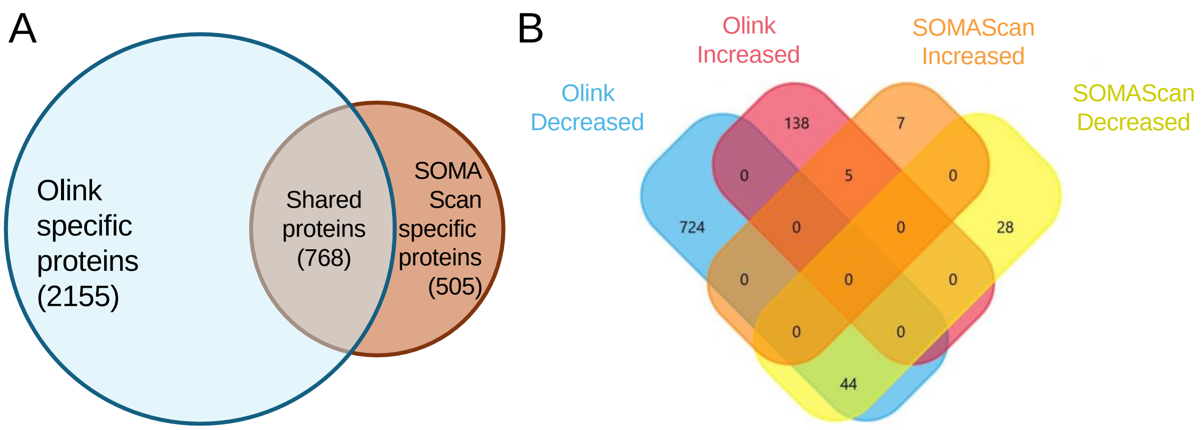

C

UniProt	SOMAScan dataset (EntrezGeneSymbol)	Fold Change (week 1 vs baseline)	Adjusted p-value	Olink dataset (HGNC symbol)	Effect (day 7 vs day -2)	p-value
P48357	LEPR	2.26	0.0028	LEPR	3.77	1.0E-25
Q03154	ACY1	2.11	0.0307	ACY1	0.90	1.5E-07
O95750	FGF19	1.82	0.0487	FGF19	1.37	2.6E-06
P10145	CXCL8	1.30	0.0418	IL8	0.52	3.7E-03
Q13093	PLA2G7	1.28	0.0386	PLA2G7	1.00	6.8E-08
P16035	TIMP2	-1.19	0.0379	TIMP2	-1.51	5.6E-06
O15197	EPHB6	-1.19	0.0379	EPHB6	-1.27	1.1E-03
Q6NW40	RGMB	-1.23	0.0374	RGMB	-1.51	4.5E-03
Q16620	NTRK2	-1.23	0.0404	NTRK2	-0.82	5.8E-03
P05546	SERPIND1	-1.24	0.0200	SERPIND1	-1.56	2.5E-13
Q9HB29	IL1RL2	-1.27	0.0170	IL1RL2	-2.05	1.1E-14
O60243	HS6ST1	-1.28	0.0108	HS6ST1	-3.06	1.5E-05
Q15582	TGFBI	-1.28	0.0417	TGFBI	-0.73	6.0E-04
P24592	IGFBP6	-1.29	0.0032	IGFBP6	-3.20	3.5E-18
P01034	CST3	-1.30	0.0174	CST3	-1.54	5.3E-09
P43652	AFM	-1.30	0.0129	AFM	-1.34	2.8E-06
P08294	SOD3	-1.32	0.0037	SOD3	-0.87	4.4E-06
P06396	GSN	-1.33	0.0002	GSN	-2.04	7.4E-06
Q03167	TGFBR3	-1.34	0.0123	TGFBR3	-1.24	4.7E-04
P08174	CD55	-1.35	0.0067	CD55	-1.00	1.1E-02
Q76M96	CCDC80	-1.35	0.0045	CCDC80	-1.89	1.5E-14
P36955	SERPINF1	-1.36	0.0016	SERPINF1	-1.56	2.1E-09
P49862	KLK7	-1.37	0.0417	KLK7	-1.97	2.8E-09
O75509	TNFRSF21	-1.37	0.0016	TNFRSF21	-1.35	1.7E-03
Q9Y240	CLEC11A	-1.39	0.0482	CLEC11A	-1.69	7.6E-10
Q96B86	RGMA	-1.41	0.0035	RGMA	-1.36	3.4E-11
Q6ZVN8	HFE2	-1.43	0.0002	HFE2	-1.56	6.8E-10
Q969Z4	RELT	-1.43	0.0143	RELT	-2.00	4.6E-08
P10912	GHR	-1.43	0.0048	GHR	-0.48	7.5E-05
P22692	IGFBP4	-1.44	0.0006	IGFBP4	-1.70	2.0E-06
P05154	SERPINA5	-1.46	0.0000	SERPINA5	-3.98	5.7E-24
Q4KMG0	CDON	-1.46	0.0049	CDON	-1.04	1.2E-09
Q8NBP7	PCSK9	-1.49	0.0079	PCSK9	-4.72	6.6E-28
O43155	FLRT2	-1.50	0.0215	FLRT2	-1.47	2.2E-06
P00736	C1R	-1.50	0.0107	C1R	-1.84	4.9E-08
Q9H773	DCTPP1	-1.50	0.0065	DCTPP1	-2.15	7.8E-10
Q01973	ROR1	-1.51	0.0001	ROR1	-1.79	3.7E-14
Q9NPY3	CD93	-1.52	0.0002	CD93	-1.05	1.4E-04
Q9NR71	ASAH2	-1.52	0.0048	ASAH2	-1.87	4.1E-19
P29622	SERPINA4	-1.52	0.0000	SERPINA4	-3.19	5.6E-20
P52799	EFNB2	-1.56	0.0207	EFNB2	-1.71	1.3E-07
O60259	KLK8	-1.59	0.0000	KLK8	-2.26	3.4E-13
Q99969	RARRES2	-1.67	0.0002	RARRES2	-2.44	9.4E-19
P35443	THBS4	-1.71	0.0000	THBS4	-2.13	1.4E-17
Q9UGM5	FETUB	-1.76	0.0001	FETUB	-3.48	3.7E-30
P22004	BMP6	-1.79	0.0017	BMP6	-1.35	4.7E-02
O15444	CCL25	-1.85	0.0061	CCL25	-0.64	7.3E-03
P28325	CST5	-1.99	0.0259	CST5	-1.30	3.8E-06
P21709	EPHA1	-2.40	0.0088	EPHA1	-5.34	1.3E-30

Supplementary Figure 5

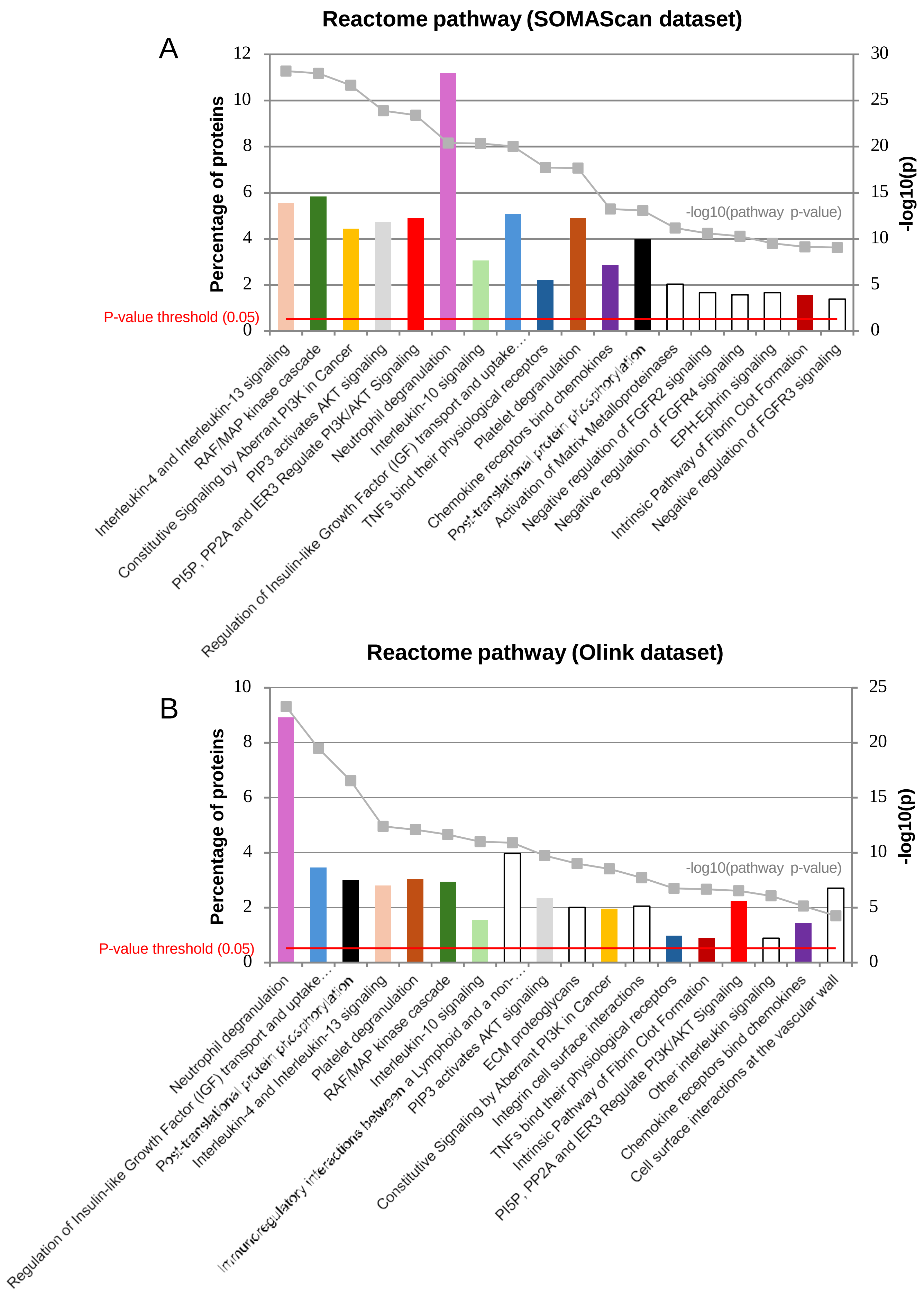

Supplementary Figure 6

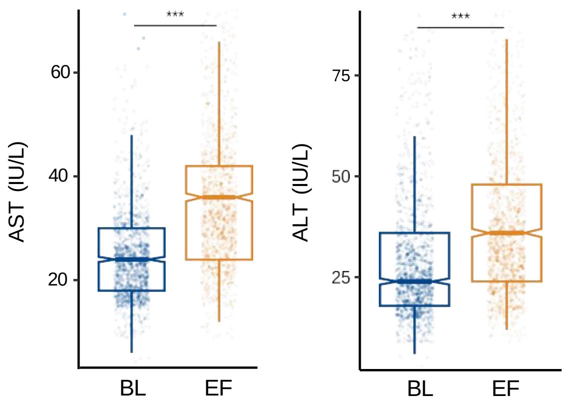

Supplementary Figure 7

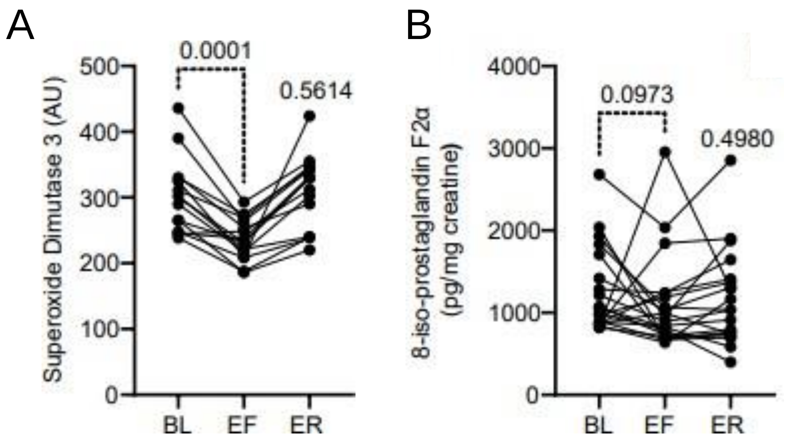

Supplementary Figure 8
