# Supplementary material for: Prolonged fasting promotes systemic inflammation and platelet activation in humans: A medically supervised, water-only fasting and refeeding study
Source: Mol Metab. 2025 Apr 21;96:102152. doi: 10.1016/j.molmet.2025.102152 (PMC12088818; doi:10.1016/j.molmet.2025.102152)
Supplement: Multimedia component 2 [file mmc2.docx]

**STAR METHODS**

**STUDY PARTICIPANT DETAILS**

The study protocol, approved by the institutional review board of the Marin General Hospital, Greenbrae, CA, USA, received written informed consent from volunteers. The study was performed per the principles in the Declaration of Helsinki. Volunteers were approached by the study team at TrueNorth Health Center, a private facility offering medically supervised fasting. The study team operated independently from the Center. Individuals at the Center were given the opportunity to volunteer for the study, and those interested were screened for eligibility. Out of 168 individuals screened for eligibility, 33 met the inclusion criteria, encompassing individuals of both genders, aged 18 or older, with a body mass index (BMI) of ≥ 20 kg/m^2^. Exclusion criteria included any history of chronic disease, physical or psychiatric conditions, use of medications incompatible with fasting, or other factors such as alcoholism or life situations that could interfere with the intervention or compliance (**Supplementary Table 1**). Twenty participants (N = 20), with a baseline BMI of 28.8±6.4 kg/m^2^ (range 21.1-50.3 kg/m^2^) consented to and commenced the medically supervised water-only fasting. Their average age was 52.2±11.8 years (range 31–72 years), with eleven being women. Detailed baseline characteristics are provided in **Table 1**. Before the study, ten individuals were not taking any medications, nine were using medications listed in Supplementary Table 2, and one used a CPAP at night. All individuals on medications (except volunteer 17, who continued using an estrogen cream) stopped their medications 1-4 days prior to the fast to allow baseline samples to be collected after the medication was discontinued. During fasting, two volunteers took medications prescribed by their doctor (fexofenadine, dichloralphenazone, acetaminophen, isometheptene) as needed, while 18 volunteers did not. During refeeding, one volunteer took medication (tamsulosin) for urinary pain. Therefore, the majority of samples were obtained without medication. However, we acknowledge that the pause in medication intake prior to baseline sample collection could have influenced the data. Fourteen individuals were non-smokers, five were former smokers, and one was a current smoker (volunteer 20, who smoked 4-5 times per week for 20 years). The former smokers include volunteer 04 (smoked for 4 years, 10 cigarettes/day, quit 10 years ago), volunteer 05 (smoked for 6 months, 1-2 cigarettes/day, quit 21 years ago), volunteer 06 (smoked for 10 years, 15 cigarettes/day, quit 19 years ago), volunteer 12 (smoked for 30 years, 10 cigarettes/day, quit 12 years ago), and volunteer 13 (smoked for 20 years, 15 cigarettes/day, quit 40 years ago). The primary objective was to determine whether PF significantly reduces inflammation, a process deeply involved in the pathogenesis of multiple age-associated chronic diseases and in the biology of aging itself. The primary outcome measure was circulating C-reactive protein (CRP) levels and other inflammatory markers at baseline and during fasting and refeeding phases.

**Medically supervised, water-only fasting and refeeding protocol.** The water-only fasting and refeeding protocol was conducted at TrueNorth Health Center in Santa Rosa, California, a specialized medical facility for prolonged water-only fasting. The protocol was administered by physicians who thoroughly examined participants' physical, neurological, and psychological health. However, all the data and measurements were independently collected by Dr. Serena Commissati from May to December 2017. Participants underwent a medical history review, urinalysis, complete blood count with differentials, and a comprehensive metabolic panel. Before fasting, participants consumed a diet of fresh raw fruits and steamed vegetables for at least two days. During fasting, participants remained at the facility, consuming a minimum of 1182 mL of water per day. Participants were instructed to avoid strenuous exercise, with only minimal physical activity (such as light walks and stretching) allowed. Medical staff closely monitored vital signs and symptoms twice daily, repeating urinalysis and blood tests weekly or as directed. Sample collection was optimized to minimize heterogeneity in fasting duration as volunteers decided the number of fasting days, ranging from 7 to 16 days. Samples were collected at baseline, fasting day 7, last day of fasting, and last day of refeeding for each participant (**Supplementary Figure 2**). Fasting was discontinued based on symptom stabilization, patient request, or medical necessity. Gradual refeeding began post-fasting, starting with juice consumption on the first day, gradually introducing solid, whole-plant foods without added sugar, oil, and salt. Moderate exercise was reintroduced gradually, with clinicians providing twice-daily monitoring during the refeeding phase.

**METHOD DETAILS**

**Anthropometrics**. Anthropometric measurements included height, measured without shoes to the nearest 0.1 cm, and body weight, obtained on a balance scale in the morning after a 12-hour fast. Body Mass Index (BMI) was calculated by dividing body weight (in kilograms) by the square of height (in meters). Waist circumference was measured to the nearest 0.1 cm at the iliac crest level during minimal respiration.

**Blood analyses**. Venous blood was sampled after an overnight fast, and processed for storage at –80°C. The Core Laboratory for Clinical Studies at Washington University in St. Louis analyzed all serum samples. Technicians, unaware of the timepoint assignment, conducted assessments. High-sensitivity C-reactive protein (hsCRP) was measured using a particle-enhanced immunoturbidimetric assay (Roche cobas c501). Commercial Enzyme-Linked Immunosorbent Assay (ELISA) Quantikine kits (R&D System Inc, Minneapolis, MN) were used for measuring other hormones.

**Proteomics.** Blood proteome was conducted on a subset of 15 individuals arbitrarily selected using the SOMAScan protein array platform at baseline (BL), during fasting (day 7 and end of fasting (EF)), and after refeeding (ER). Plasma samples were processed by SomaLogics technicians following recommended standard protocols for SOMAScan Assay Human Plasma, as described elsewhere.(49, 50) A SOMAScan Quality Statement was provided for normalization and calibration. The assay profiled 1,317 protein analytes.

**Plasma Aβ42 and Aβ40 quantitation.**  This immunoprecipitation liquid chromatography-tandem mass spectrometry (IP-LC-MS/MS) assay has been clinically and analytically validated (15-17, 19) and is performed in a CLIA-certified, CAP-accredited, ISO 13485-compliant laboratory at C_2_N Diagnostics (St. Louis, MO, USA). Briefly, plasma samples are spiked with known quantities of stable isotope labeled internal standard Aβ42 and Aβ40 proteins (r-Peptide, Watkinsville, GA), plasma Aβ isoforms are immunoprecipitated, enzymatically digested into Aβ42- and Aβ40-specific peptides that are separated using micro-flow liquid chromatography (Waters Corp., Milford, MA, USA), and sprayed into the source of a Fusion Lumos mass spectrometer (Thermo Fisher Scientific, Waltham, MA). Fragment ions formed from endogenous and exogenous Aβ42 and Aβ40 peptides are monitored, their peak areas are quantified and compared to the same peak areas monitored in a series of four calibrators formulated in human serum albumin, processed and analysed (as above) in parallel with the human plasma samples. In plasma, the peak area ratios for endogenous and internal standard peptides are compared to the same for the calibration standard curve, and plasma Aβ42 and Aβ40 concentrations are obtained from the respective standard curve. Plasma Aβ42/40 ratio is calculated by dividing the plasma Aβ42 concentration by the Aβ40 concentration (both in pg/mL).

**Urine measurements.** The 11-dehydro-thromboxane(TX)B_2_ is one of the major urinary enzymatic metabolite of TXA_2_/TXB_2_ in humans and an index of *in vivo* platelet activation(22). The urinary 8-iso-prostaglandin (PG)F_2α_ is a non-enzymatic, oxidation product of arachidonic acid and an *in vivo* biomarker of lipid peroxidation(51). Briefly, urine samples were thawed, centrifuged, 2000 cpm of ^3^H-PGE_2_ (3.70-6.86 TBq/mmol, Perkin Elmer, Boston, USA) were added to 1 mL-urine samples that were loaded onto a 1 mL/50 mg C18 column (BakerbondTM-spe, J.T.Baker, Gliwice, Poland) and eluted with 2.5 mL of isooctane/ethyl acetate (1:1, vol/vol). The eluate was then transferred to a 1 mL/100 mg SiOH column (BakerbondTM-spe, J.T.Baker) and eluted with 2 mL of ethyl acetate/methanol (60:40, vol/vol), dried and resuspended in 1 mL of PBS/0.1% BSA buffer for subsequent immunoassay and recovery count. Biomarkers were measured with a standard ELISA as previously described(52) using specific antibodies(53). Urinary creatinine was measured with a commercial kit (Creatinine Colorimetric Detection Kit; Enzo Life Sciences, Farmingdale, NY). The final value of each biomarker was corrected for the percentage of recovery based on the ^3^H-PGE_2_ cpm and expressed as pg/mg of creatinine.

**Buchinger-Wilhelmi Clinic study participants**. The Buchinger-Wilhelmi Clinic (BWC) study, as outlined by Wilhelmi de Toledo et al. in 2019,(5) involved 1422 participants aged 18-99 years. Ethics approval for the original study (German Clinical Trials Register ID: DRKS00010111) was obtained by Wilhelmi de Toledo et al., with approval granted by the medical council of Baden-Württemberg and the Ethics Committee of the Charité-University Medical Center, Berlin. The original study was performed following the Declaration of Helsinki and written informed consent was obtained from participants. The individuals, without predefined contraindications to Buchinger fasting, voluntarily joined the clinic for preventive or therapeutic fasting. Fasting durations ranged from 4 to 21 days, with participants categorized into fasting lengths of 5, 10, 15, and 20 days for analysis. Fasting guidelines included a daily intake of 200–250 kcal and 25–35 g of carbohydrates, obtained from fruit juice and vegetable soup, along with 3 liters of water or non-caloric herbal teas. This study independently analyzed published, publicly available data from the original study.

**QUANTIFICATION AND STATISTICAL ANALYSIS**

**Statistical analysis.** The SOMAScan proteomics dataset included N = 15 participants at baseline, fasting day 7, and last day of fasting and refeeding. All other assays reported in this manuscript included N = 20 participants. Statistical analyses for all variables were performed using pairwise differences between baseline, fasting (either day 7 or last day of fasting), and last day of refeeding assessed using Student’s t-test (2 timepoints, normal data), Wilcoxon signed-rank test (non-normally distributed data), and one-way ANOVA (3 timepoints). Changes were expressed as absolute quantities, relative quantities normalized to baseline, or fold-change (FC) relative to baseline. All statistical tests were two-tailed, and significance was considered at adjusted p-value < 0.05, unless specified otherwise. The same statistical approach was applied to analyze data from the previously published Buchinger-Wilhelmi cohort. Data were analyzed and visualizations were produced with GraphPad Prism (version 10), QIAGEN IPA (QIAGEN Inc., <https://digitalinsights.qiagen.com/IPA>, accessed October 2024), FunRich: Functional Enrichment analysis tool (version 3.1.4, [FunRich :: Functional Enrichment Analysis Tool :: Home](http://www.funrich.org/index.html)), and Excel.

The correlation between BHB, CRP, and vWF with the circulating SOMAScan proteome was examined using mixed-effect regression models for longitudinal data, with individuals as the random effect. A False Discovery Rate (FDR) correction for multiple testing was applied to control for the type I error rate. Pathway enrichment analyses utilized the pathfindR package,(54) mapping significant (p < 0.01) proteins into active sub-networks based on the reference protein-protein interaction database. The active subnetworks were filtered based on the number of significant genes and their interaction likelihood scores. Finally, the list of subnetworks was used as the input for the enrichment analyses. We used the BioGrid (https://thebiogrid.org/) database as the reference for protein-protein interactions, and the KEGG database (https://www.genome.jp/kegg/) for biological pathways. We defined pathways with FDR-adjusted p < 0.01 as statistically significant. All analyses and graphs were performed using the open-source R software version 4.2.1 and RStudio.

**Ingenuity Pathway Analysis.** Alterations in canonical pathways were generated with IPA software (QIAGEN Inc., https://www.qiagenbio informatics.com/products/ingenuity-pathway-analysis, October 2024) using the SOMAScan proteomics dataset as an input (n = 1,317 proteins), including protein identifiers (Uniprot), fold-changes (fasting/baseline), and adjusted p-values. IPA mapped 1,255 entities (1,255/1,317 = 95%) that were analyzed using Core Analysis Expression Analysis based on log2(FC) values. The adjusted p-value cutoff was 0.05, producing 82 analysis-ready molecules (71 downregulated and 11 upregulated). The full list of significantly altered canonical pathways and details of synaptogenesis and amyloid fiber formation are presented in Supplementary Material.

**Reactome pathway analysis**. Alterations in Reactome pathways were generated with FunRich software ([FunRich :: Functional Enrichment Analysis Tool :: Home](http://www.funrich.org/), October 2024) using the SOMAScan proteomics dataset (n = 1,255 proteins) as an input, including protein identifiers (Uniprot), fold-changes (fasting/baseline), and adjusted p-values. The Reactome pathways database mapped 1,081 proteins and calculated the percentage of proteins per pathway (No. of genes in the dataset divided by No. of genes in the Uniprot background dataset) and the -log10(adjusted p-value). Significance threshold < 0.05.

References

[48] Candia J, Cheung F, Kotliarov Y, Fantoni G, Sellers B, Griesman T, et al. Assessment of variability in the SOMAscan assay. Sci Rep. 2017;7(1):14248.

[49] Kim CH, Tworoger SS, Stampfer MJ, Dillon ST, Gu X, Sawyer SJ, et al. Stability and reproducibility of proteomic profiles measured with an aptamer-based platform. Sci Rep. 2018;8(1):8382.

[50] Petrucci G, Rizzi A, Hatem D, Tosti G, Rocca B, Pitocco D. Role of oxidative stress in the pathogenesis of atherothrombotic diseases. Antioxidants. 2022;11(7).

[51] Lellouche F, Fradin A, Fitzgerald G, Maclouf J. Enzyme immunoassay measurement of the urinary metabolites of thromboxane A2 and prostacyclin. Prostaglandins. 1990;40(3):297-310.

[52] Pradelles P, Grassi J, Maclouf J. Enzyme immunoassays of eicosanoids using acetylcholine esterase as label: an alternative to radioimmunoassay. Anal Chem. 1985;57(7):1170-1173.

[53] Ulgen E, Ozisik O, Sezerman OU. pathfindR: an R package for comprehensive identification of enriched pathways in omics data through active subnetworks. Front Genet. 2019;10:858.
